# Antiglycation Activity of Aucubin In Vitro and in Exogenous Methylglyoxal Injected Rats

**DOI:** 10.3390/molecules24203653

**Published:** 2019-10-10

**Authors:** Eunsoo Jung, Su-Bin Park, Woo Kwon Jung, Hyung Rae Kim, Junghyun Kim

**Affiliations:** 1 Laboratory of Toxicology, Research Institute for Veterinary Science and College of Veterinary Medicine, Seoul National University, Seoul 08826, Korea; ozz79@snu.ac.kr; 2 Department of Oral Pathology, School of Dentistry, Chonbuk National University, Jeonju 54896, Korea; tnqls309@gmail.com (S.-B.P.); wkjungjbnu@gmail.com (W.K.J.); rlagudfo31@gmail.com (H.R.K.)

**Keywords:** advanced glycation end products, aucubin, methylglyoxal

## Abstract

Advanced glycation end products (AGEs) is a causative factor of various chronic diseases, including chronic kidney disease and atherosclerosis. AGE inhibitors, such as aminoguanidine and pyridoxamine, have the therapeutic activities for reversing the increase in AGEs burden. This study evaluated the inhibitory effects of aucubin on the formation of methylglyoxal (MGO)-modified AGEs in vitro. We also determined the potential activity of aucubin in reducing the AGEs burden in the kidney, blood vessel, heart, and retina of exogenously MGO-injected rats. Aucubin inhibited the formation of MGO-modified AGE-bovine serum albumin (IC_50_ = 0.57 ± 0.04 mmol/L) and its cross-links to collagen (IC_50_ = 0.55 ± 0.02 mmol/L) in a dose-dependent manner. In addition, aucubin directly trapped MGO (IC_50_ = 0.22 ± 0.01 mmol/L) in vitro. In exogenous MGO-injected rats, aucubin suppressed the formation of circulating AGEs and its accumulation in various tissues. These activities of aucubin on the MGO-derived AGEs in vitro and in vivo showed its pharmacological potential for inhibiting AGEs-related various chronic diseases.

## 1. Introduction

The advanced glycation end products (AGEs) are generated in the human body and affect the structure and function of proteins. This process was first introduced by Louis-Camille Maillard in 1912 [1]. The glycation process leads to loss of protein function and impaired tissue elasticity [2,3,4]. AGEs are slowly formed during the process of aging, and some conditions such as diabetes and tissue oxidative stress accelerate this process [5,6]. AGEs are naturally formed in trace amounts in the body by protein or lipid glycation with sugars, and most of them are catabolized depending on the state or type of antioxidative systems, in respective tissues, macromolecular turnover, receptor-mediated degradation, and renal elimination [7]. However, a chronic increase in intracellular oxidative stress accelerates the formation of AGEs and leads to their accumulation in the intracellular space. Oxidative stress is further fueled by excessive reactive oxygen stress (ROS) generation from glucose autoxidation and also the nonenzymatic, covalent attachment of glucose molecules to circulating proteins that result in the formation of AGEs [8,9,10]. The formation of AGEs is an irreversible reaction and AGEs can form cross-links with proteins resulting in disturbed biochemical reactions, thus AGEs are implicated in pathogenic processes of various age-related diseases [11]. Particularly, matrix proteins such as collagen are properly cross-linked with AGEs in conditions like diabetes and aging [12,13].

Methylglyoxal (MGO) is known as a major precursor of AGEs and is generated as a by-product of glycolysis. MGO easily forms AGEs due to its high reactivity which allows it to cross-link with proteins [14]. MGO-derived protein modifications have been shown in human tissues [15]. Previous studies have shown that AGEs play an important role in pathogenic processes involved in chronic kidney disease (CKD) [16], Alzheimer’s disease [17], and diabetic complications [18].

Some natural and synthetic compounds have been considered to be AGE inhibitors [19]. *Aucuba japonica* Thunb. is a traditional Korean medicinal herb and has been used to treat several diseases, such as edema and inflammation [20]. Aucubin is an iridoid glucoside isolated from this plant and has various pharmacological activities, such as antioxidant, anti-inflammatory, antimicrobial, antianalgesic, and antitumor effects [21,22,23]. Despite the various effects of *A. japonica* and its bioactive ingredient aucubin, it remains unclear whether aucubin has inhibitory effects on the glycation processes and its cross-links with proteins. Therefore, the aim of this study was to evaluate the inhibitory effect of aucubin on the formation of MGO-derived AGEs in vitro; furthermore, aucubin was used in exogenous MGO-injected rats to verify its preventive effect on the accumulation of AGEs in vivo.

## 2. Results

### 2.1. Inhibitory Activity of Aucubin on MGO-Derived AGEs Formation In Vitro

**Figure 1:**
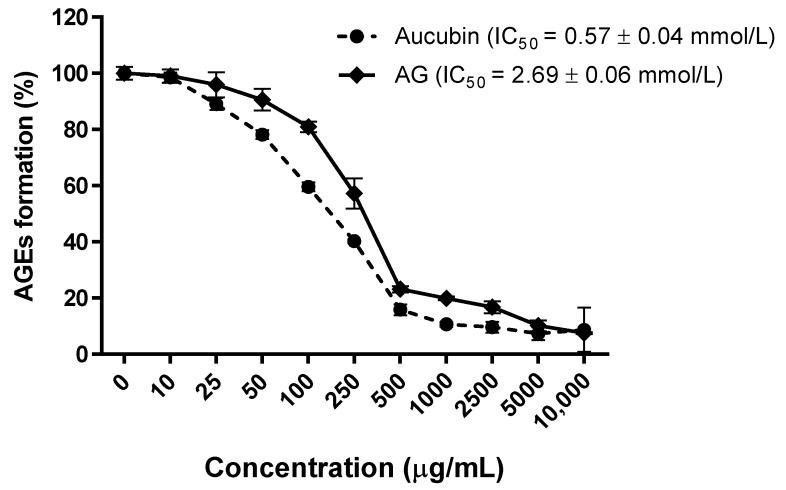
Inhibitory effect of aucubin and AG on the formation of methylglyoxal (MGO)-derived advanced glycation end products (AGEs) in vitro. All results are expressed as the mean ± SE, *n* = 4. The IC_50_ values were determined from the plotted graph.

As shown in Figure 1, aucubin exhibited inhibitory activity on the formation of MGO-derived AGE (IC_50_ = 0.57 ± 0.04 mmol/L), its inhibitory activity was 5-times stronger than AG (IC_50_ = 2.69 ± 0.06 mmol/L).

### 2.2. Inhibitory Activity of Aucubin on AGEs Cross-Linking with Rat Tail Tendon Collagen

**Figure 2:**
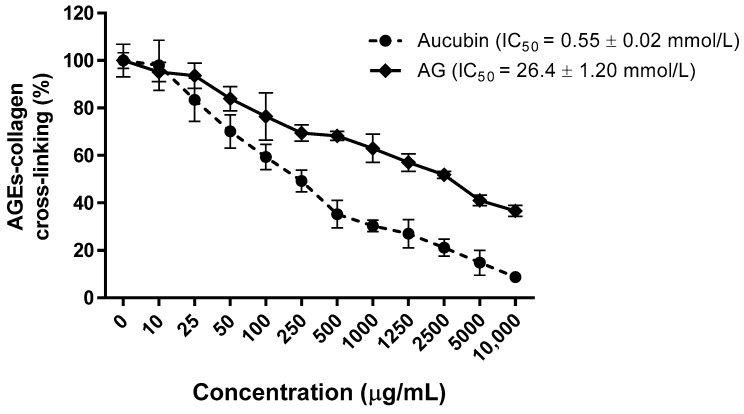
Inhibitory effect of aucubin and AG on the cross-links of AGEs with collagen in vitro. All results are expressed as the mean ± SE, *n* = 4. The IC_50_ values were determined from the plotted graph.

The inhibition of AGE-BSA cross-linking to collagen at various concentrations of aucubin was tested. As shown in Figure 2, aucubin inhibited dose-dependently the cross-linking of AGE-modified BSA with collagen (IC_50_ = 0.55 ± 0.02 mmol/L) and has a 48-times stronger antiglycation activity than AG (IC_50_ = 26.40 ± 1.20 mmol/L).

### 2.3. Methylglyoxal Breaking Effect of Aucubin

**Figure 3:**
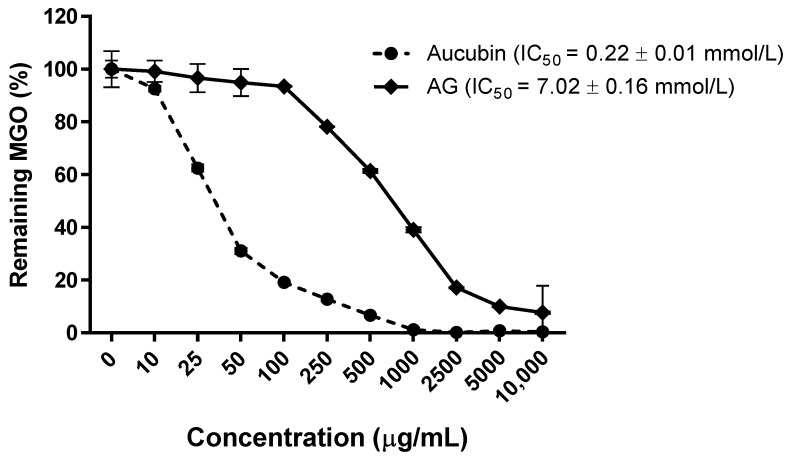
MGO breaking activity of aucubin and AG. All results are expressed as the mean ± SE, *n* = 4. The IC_50_ values were determined from the plotted graph.

To investigate the role of aucubin as a potential AGE inhibitor, we tested whether aucubin can break MGO in vitro. As shown in Figure 3, aucubin broke dose-dependently MGO (IC_50_ = 0.22 ± 0.01 mmol/L) and its activity was 32-times stronger than AG (IC_50_ = 7.02 ± 0.16 mmol/L).

### 2.4. Effect of Aucubin on AGEs Formation in Exogenous MGO-Injected Rats

**Figure 4:**
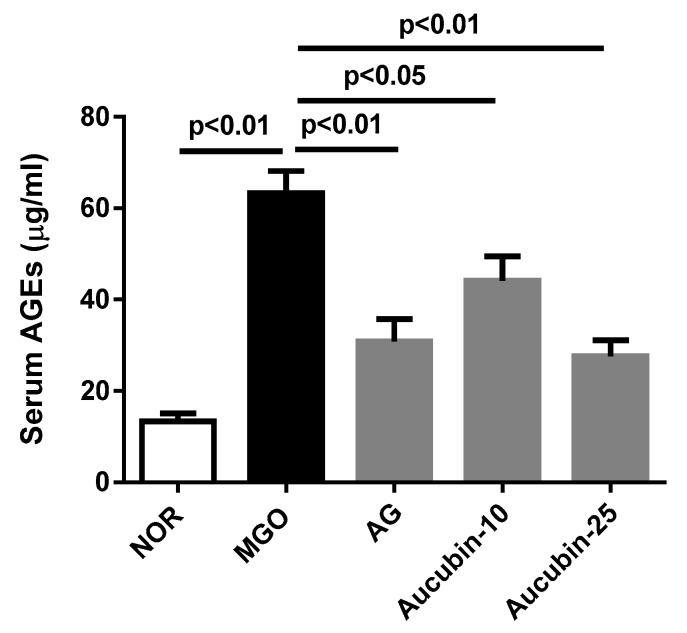
Circulating AGEs formation in the blood of exogenous MGO-injected rats. NOR, normal control rats; MGO, exogenous MGO-injected rats; AG, MGO treated with aminoguanidine (50 mg/kg); Aucubin-10, MGO treated with aucubin (10 mg/kg); Aucubin-25, MGO treated with aucubin (25 mg/kg). All data are expressed the mean ± SE, *n* = 6.

In order to determine whether intraperitoneal injection of exogenous MGO accelerates the formation of MGO-derived AGEs, we measured the circulating levels of AGEs in the blood. At the end of the study, AGEs were rarely found in the control group, but higher levels of those were found in the MGO-injected rats. However, the treatment of aucubin dose-dependently inhibited the formation of AGEs compared to the MGO group. Treatment with a high dose of aucubin (25 mg/kg) showed similar efficacy of inhibition as that shown by treatment with AG (50 mg/kg) (Figure 4).

### 2.5. Effect of Aucubin on AGEs Accumulations in Exogenous MGO-Injected Rats

**Figure 5:**
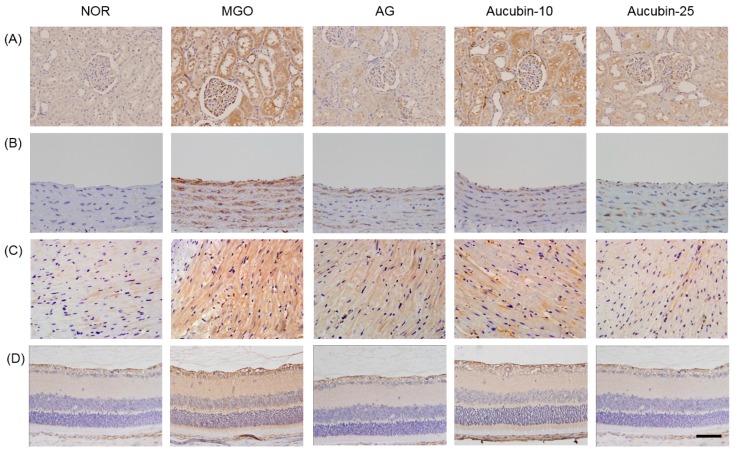

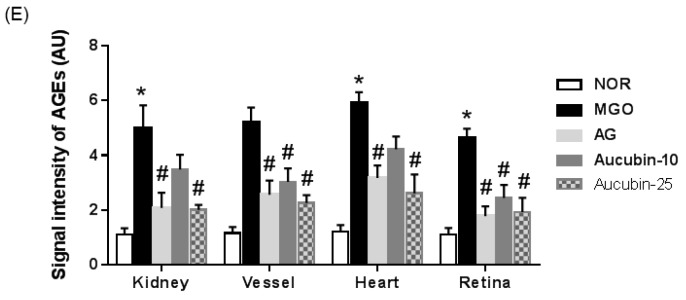
AGEs accumulation in the kidney (**A**), blood vessel (**B**), heart (**C**), and retina (**D**) of exogenous MGO-injected rats. Immunohistochemistry of AGEs. X400 magnification. NOR, normal control rats; MGO, exogenous MGO-injected rats; AG, MGO treated with aminoguanidine (50 mg/kg); Aucubin-10, MGO treated with aucubin (10 mg/kg); Aucubin-25, MGO treated with aucubin (25 mg/kg). Scale bar = 100 μm. (**E**) The intensity of immunohistochemistry was quantified. All data are expressed the mean ± SE, *n* = 6. * *p* < 0.01 vs. NOR group, ^#^ *p* < 0.01 vs. MGO group. AU: arbitrary unit.

We next determined whether the AGEs are accumulated in various tissues of the MGO-injected rats. Hence, the immunohistochemical staining of AGEs was performed. As shown in Figure 5, AGE was almost undetectable in the control group, but higher levels of those were found in kidney, blood vessel, heart, and retina of MGO injected rats. However, the treatment of aucubin dose-dependently inhibited the tissue accumulation of AGEs compared to the MGO group.

## 3. Discussion

Previous studies have revealed that AGEs play a crucial role in pathogenic processes of various diseases including Alzheimer’s disease, cardiovascular diseases, and diabetes [24,25,26,27]. AGE cross-linking forms irreversible complexes when a sugar permanently binds to a target protein, such as elastin and/or collagen. In this study, we showed that aucubin exhibits an antiglycation activity in vitro, and we also found that MGO, a reactive carbonyl, is broken by aucubin. Aucubin showed a more potent antiglycation activity than AG. Moreover, Aucubin showed preventive activity against the formation of circulating AGEs in blood and its accumulation in kidney, blood vessel, heart, and retina of rats injected with exogenous MGO.

Many studies have demonstrated that AGEs accumulate in many tissues in patients with diabetes. Their toxic effects in diabetic conditions have been demonstrated in several experimental studies [11]. MGO is a reactive carbonyl compound and a potent precursor of AGEs [28]. In particular, MGO-derived AGEs in human plasma contribute to the development of various diseases such as diabetes [14], cancer [29], and cardiovascular diseases [26]. There is no enzyme known in humans that can abolish the structure of AGEs. Thus, AGEs are easily accumulated during the process of aging [30]. The inhibition of MGO-derived AGEs and cross-linking of AGEs with protein can be an effective strategy for the prevention of age-related diseases. Reduction of MGO is known as a pharmacological strategy used to inhibit the formation of MGO-derived AGEs [31]. AG, as a well-known AGE inhibitor, prevents the accumulation of AGEs by interacting with these reactive carbonyls. Other AGE inhibitors, including 2-isopropylidenehydrazono-4-oxo-thiazolidin-5-ylacetanilide (OPB-9195) and pyridoxamine, also inhibit formation of AGEs through interaction with reactive carbonyls such as MGO scavengers [32]. This study reveals that aucubin exhibits the breaking ability of MGO. Aminoguanidine’s anti-AGE activity involves its ability to act as a scavenger of highly reactive dicarbonyls [33]. Aminoguanidine reacts with methylglyoxal to form triazine adducts [34]. Aucubin is one of the iridoid glucosides. When compared to aminoguanidine, iridoids do not form triazine adducts, as they contain no nitrogen [35]. The antiglycation action of iridoids may even involve different Maillard products. The exact mechanism of action still requires further investigation. Collectively, these results suggest that the MGO breaking activity shown by aucubin might contribute to the inhibition of both the formation and accumulation of AGEs in various tissues and might inhibit the development of AGEs-related diseases. 

In order to verify the antiglycation activity of aucubin in vivo, aucubin was administered orally to rats injected with exogenous MGO. In the MGO-injected rats, AGEs were highly generated in blood and accumulated in various tissues. AGEs are considered to play an important role in major pathogenic processes like age-related renal failure, atherosclerosis, and diabetic retinopathy [13,16,18,36,37]. In this study, aucubin prevented the formation of circulating AGEs in the blood of rats injected intraperitoneally with exogenous MGO. In addition, aucubin ameliorated the accumulation of AGEs in various tissues in these rats. Many studies have demonstrated the effects of aucubin against diabetes-induced pancreas injury [38] and carbon tetrachloride-induced hepatic damage [39] through its antioxidant action in various experimental models. MGO has been shown to induce ROS generation [40]. AGEs also act as a source of generation of ROS [13]. In our in vitro system, the IC_50_ dose of aucubin to the glycation process was as low as 20 μmol/L. Aucubin was also effective in vivo at a dose of 25 mg/kg. Xu et al. reported that the peak plasma concentration of aucubin following the oral consumption of 50 mg/kg aucubin in male SD rats was 11.6 μmol/L at a concentration peak time of 1.08 h [41]. Based on previous reports and our results, our finding regarding the effective dose of aucubin in vitro is consistent with the in vivo results. Although aucubin has shown multiple pharmacological effects, no information is available about the toxicology of aucubin. Shen et al. showed that the intraperitoneal injection of 40 mg/kg aucubin produced no toxic and adverse effects in mice [42]. Taken together, these results indicate that aucubin is able to prevent AGEs burden in various tissues by its ability to act as a breaking agent for MGO.

In conclusion, our results suggest that aucubin could be considered as a prospective candidate for preventative treatment for pathogenic processes of various chronic diseases associated with the accumulation of AGEs.

## 4. Materials and Methods

### 4.1. In Vitro Assay of the Formation of MGO-Derived AGEs

Bovine serum albumin (BSA, 10 mg/mL, Sigma Chemicals, St. Louis, MO, USA) was incubated at 4 °C for 1 week with 5 mmol/L MGO in sodium phosphate buffer (0.1 M, pH 7.4). Aucubin (Sigma Chemicals, MO, USA) and aminoguanidine (Sigma Chemicals, MO, USA) were added in this reaction mixture. The levels of MGO-derived AGEs were calculated by measuring the fluorescence intensity using a spectrofluorometer (excitation wavelength at 370 nm and emission wavelength at 440 nm). The IC_50_ concentration was calculated.

### 4.2. In Vitro Assay of the Cross-Linking of Glycated Proteins

AGE-modified BSA (1 μg, TransGenic Inc, Kobe, Japan) was treated with or without aucubin or AG, respectively, in collagen-coated 96-well plates and incubated for 4 h. The collagen-AGE-BSA cross-linked complex was detected using a horseradish peroxidase-linked mouse anti-AGE antibody (6D12, Wako, Osaka, Japan). The IC_50_ concentration was calculated.

### 4.3. MGO Assay

MGO (0.05 mmol/L, Sigma, MO, USA) was treated with or without aucubin or AG for 30 min. After incubation, o-phenylene diamine (derivatization agent, 10 mmol/L) was added to each sample, and the mixtures were kept at room temperature for 30 min for derivatization of the remaining MGO. Levels of remaining MGO were then measured as previously described [43].

### 4.4. Animals and Experimental Design

All procedures involving the use of animals were approved by the Institutional Animal Care and Use Committee (IACUC approval No. 2019-17). Sprague–Dawley (SD) rats (~200 g) were randomized into five groups of 6 rats each. Group 1: normal rat (NOR); Group 2: rats treated with MGO using an intraperitoneal (i.p.) injection (MGO); Group 3: rats treated with MGO using an i.p. injection and orally administered 50 mg/kg of AG (AG); Group 4: ~5 rats treated with MGO using an i.p. injection and orally administered two different doses of 10 and 25 mg/kg of aucubin once a day for 2 weeks. We selected the i.p. route to achieve a pathologically relevant plasma concentration of 2–5 mmol/L MGO [44,45]. Following the protocol from a previous report [46], we administered 17.25 mg/kg (240 mmol/kg) of MGO using a single i.p. injection.

### 4.5. Quantification of Serum AGE Levels

At necropsy, blood was collected and serum AGE levels were calculated using an AGE ELISA kit (MyBioSource Inc, San Diego, CA, USA) according to the manufacturer’s instruction.

### 4.6. Immunohistochemical Staining

At necropsy, the kidney, blood vessel, heart, and retina were fixed with 10% (*v*/*v*) formaldehyde and embedded in paraffin, and 5-μm-thick sections were prepared. Immunohistochemical staining was performed for AGEs using a monoclonal mouse anti-AGE antibody (6D12, Wako, Osaka, Japan) according to a previously reported method [47]. Briefly, deparaffinized sections were hydrated and treated with 1% H_2_O_2_ in methanol prior to incubation with primary antibodies for 1 h at room temperature. Signals for AGEs were detected using the Envision kit (DAKO, Carpinteria, CA, USA) and visualized using a DAB peroxidase substrate kit (Vector Labs, Burlingame, CA, USA). As a negative control, tissue sections were incubated with serum from nonimmunized animals, instead of the primary antibody. The intensity of positively stained areas was determined from five randomly selected areas at 100x magnification using the ImageJ software (National Institutes of Health, Bethesda, MD, USA).

### 4.7. Statistical Analysis

All data were expressed as the mean ± standard error of the mean (SE). Differences between groups were determined by one-way ANOVA followed by Tukey’s post-hoc test using Prism 6.0 (Graphpad, La Jolla, CA, USA).
